# A rapid detection system for core virulence and resistance genes in hypervirulent *Klebsiella pneumoniae* using multiplex fluorescence PCR-capillary electrophoresis

**DOI:** 10.3389/fmicb.2026.1798786

**Published:** 2026-04-20

**Authors:** Huimin Ning, Li Qin, Chaoqun Wang, Kai Zhou, Tiantian Wang, Qiang Feng, Wenyang Wang

**Affiliations:** 1 Department of Clinical Laboratory, The Affiliated Taian City Central Hospital of Qingdao University, Tai'an, China; 2 Joint National-Local Engineering Research Centre for Safe and Precise Coal Mining, Huainan, China

**Keywords:** antibiotic resistance genes, capillary electrophoresis, hypervirulent *Klebsiella pneumoniae*, multiplex fluorescence PCR, rapid detection, virulence genes

## Abstract

Infections caused by hypervirulent *Klebsiella pneumoniae* (hvKp) have increased in clinical settings, yet rapid methods for integrated virulence–serotype–resistance profiling remain underdeveloped. In this study, we developed a multiplex fluorescence PCR-capillary electrophoresis (MPCE) system for the rapid and simultaneous detection of core genetic markers in hvKp. Specifically, the MPCE assay detected 16 genetic markers: five virulence genes (*iucA*, *iroB*, *peg344*, *rmpA*, and *rmpA2*), two major capsular serotype genes (K1 and K2), seven resistance genes (*blaKPC*, blaNDM, *blaCTX-M*, *blaSHV*, *blaOXA-23*, *blaOXA-48* and *mcr-1*), and two internal controls. Amplicons were subsequently analyzed via capillary electrophoresis and GeneMapper software. As a result, the MPCE system simultaneously detected all 16 targets in 152 min, demonstrating single-base resolution that enabled precise discrimination between closely related amplicons. Moreover, the assay achieved a limit of detection of 10^2^ copies/μL, exhibited excellent repeatability, and showed no cross-reactivity against a panel of non-target pathogens. Furthermore, clinical validation confirmed its strong concordance with next-generation sequencing (*κ* = 0.659–1.000). Therefore, the MPCE-based assay provides a high-throughput, sensitive, and specific platform that enables simultaneous profiling of virulence and resistance genes for the comprehensive genotyping of hvKp. It represents a valuable tool for enhancing antimicrobial resistance surveillance and epidemiological investigations.

## 1 Introduction

*Klebsiella pneumoniae* is a formidable Gram-negative pathogen responsible for a wide range of hospital- and community-acquired infections, including pneumonia, bacteremia, and pyogenic liver abscesses (Wyres et al., 2020). Recently, the global healthcare landscape has been further complicated by the rapid dissemination of a hypervirulent variant, hypervirulent *Klebsiella pneumoniae* (hvKp), which exhibits distinct epidemiological and clinical characteristics compared to classical *Klebsiella pneumoniae* (cKp) (Al Ismail et al., 2025; Choby et al., 2019). Unlike cKp, which predominantly affects immunocompromised hosts, hvKp can cause severe invasive infections in healthy, immunocompetent individuals. These infections are frequently characterized by metastatic dissemination to distant sites, including the eyes, central nervous system, and joints, with mortality rates from hvKp-associated bacteremia exceeding 30% in certain cohorts (Li et al., 2017; Lan et al., 2020). A more concerning development is the convergence of hypervirulence and carbapenem resistance. The horizontal acquisition of plasmid-encoded carbapenemase genes (*blaKPC* and *blaNDM*) by hvKp clones has led to the emergence of multidrug-resistant hypervirulent (MDR-hvKp) strains. These strains effectively evade last-line antibiotics, including carbapenems, resulting in severely limited therapeutic options and poor clinical outcomes (Tang et al., 2020).

The hypermucoviscous phenotype, traditionally assessed using the string test, is a hallmark of hvKp (Fang et al., 2004). However, this method demonstrates limited clinical reliability. Up to 15–20% of genetically confirmed hvKp isolates do not exhibit hypermucoviscosity, and false-negative rates exceed 40% in immunocompromised patients (Lee et al., 2006; Lin et al., 2011; Catalán-Nájera et al., 2017). Moreover, conventional diagnostic workflows-including culture, phenotypic identification, virulence assays, and antimicrobial susceptibility profiling-typically require 3–5 days, far exceeding the critical 48-h window necessary for effective intervention in MDR-hvKp-associated sepsis. Molecular diagnostic approaches have identified key genetic determinants of hvKp virulence, including capsule overexpression regulators (*rmpA* and *rmpA2*), siderophore-related genes (*iucA* and *iroB*), and the virulence-associated gene *peg344* (Russo et al., 2018; Xu et al., 2023). These genes display serotype-specific distribution patterns; for instance, the K1 serotype carries these virulence determinants at frequencies exceeding 95%. Notably, siderophore expression correlates quantitatively with serum resistance and *in vivo* bacterial burden, reinforcing the use of genetic markers as reliable proxies for pathogenic potential (Russo et al., 2018; Russo et al., 2021).

Currently, the hvKp detection primarily relies on molecular biological techniques, which encounter several significant limitations. Conventional PCR assays are restricted to a limited number of targets per reaction, hindering the simultaneous detection of key virulence (*peg344* and *iroB*) and resistance genes (*blaOXA-48*, *blaKPC*, and *mcr-1*) (Yan et al., 2023). Multiplex PCR assays are further constrained by reduced sensitivity and false negatives arising from primer interference and amplicon competition, particularly when detecting low-abundance targets such as the K2 capsular gene (Wan et al., 2023). Although next-generation sequencing (NGS) offers comprehensive genotyping capability, its long turnaround time and high cost hinder its applicability for routine clinical diagnostics. Consequently, there is an urgent need for a high-throughput, rapid, and accurate assay that integrates both virulence and resistance profiling.

To address this gap, we developed a multiplex fluorescence PCR–capillary electrophoresis (MPCE) assay that enables comprehensive hvKp genotyping. This platform employs 14 fluorescently labeled primers to concurrently detect five core virulence genes (*iucA, iroB, peg344, rmpA*, and *rmpA2*), two capsular serotypes (K1 and K2), and seven resistance genes (*blaKPC, blaNDM, blaCTX-M, blaSHV, blaOXA-23*, *blaOXA-48* and *mcr-1*). Capillary electrophoresis achieves single-base resolution for amplicons ranging from 50 to 400 bp, enabling precise discrimination between closely related targets such as *rmpA* and *rmpA2*. By enabling rapid, concurrent virulence–resistance gene profiling, this integrated system represents a valuable tool for enhancing antimicrobial resistance surveillance and epidemiological investigations of hvKp.

## 2 Materials and methods

### 2.1 Target gene selection and primer design

Target genes for the MPCE assay were selected based on a comprehensive literature review and genomic databases (NCBI GenBank). The panel included five core virulence genes of hvKp (*iucA, iroB, peg344, rmpA, and rmpA2*) (Russo et al., 2024; Tang et al., 2025), two capsular serotype marker genes (K1 and K2) (Harada and Doi, 2018), seven common antibiotic resistance genes (*blaKPC, blaNDM, blaCTX-M, blaSHV, blaOXA-23, blaOXA-48,* and *mcr-1*), and two internal control genes (*rcsA* for *K. pneumoniae* and IC for system quality control) (Al Ismail et al., 2025; Wang et al., 2020). Primers were designed based on published sequences and analyzed using DNAMAN software (Lynnon Biosoft) to ensure amplification of fragments ranging from 50 to 400 bp. The forward primers were synthesized with a 5’-FAM fluorescent label (Supplementary Table S1). Primer specificity was confirmed by in silico analysis using BLAST, and all primers were synthesized by Sangon Biotech (Shanghai, China).

### 2.2 DNA extraction and quality control

Bacterial genomic DNA was extracted using the QIAamp DNA Mini Kit (Qiagen, Germany) following the manufacturer’s instructions. The concentration and purity of the extracted DNA were assessed using a NanoDrop 2000 spectrophotometer (Thermo Fisher Scientific, USA). All samples exhibited A260/A280 ratios between 1.8 and 2.0, indicating high DNA purity. The extracted DNA samples were stored at −20 °C for subsequent use.

### 2.3 Optimization of the multiplex PCR amplification system

The multiplex PCR reaction was performed in a 25 μL volume containing 6.25 μL of C199P1 multiplex PCR master mix, 6 μL of primer mixture (with each primer adjusted to a final concentration of 300 nM), 2 μL of template DNA (50–100 ng), and nuclease-free water to make up the remaining volume. The amplification protocol consisted of an initial denaturation at 95 °C for 5 min followed by 35 cycles of denaturation at 95 °C for 30 s, annealing at 60 °C for 30 s, and extension at 72 °C for 30 s, with a final extension step at 72 °C for 5 min.

Amplification products were initially evaluated by 2% agarose gel electrophoresis and subsequently separated via capillary electrophoresis using an ABI-3730-XL Genetic Analyzer (Applied Biosystems, USA). Electropherogram peaks were analyzed using GeneMapper software (version 5.0) with default analytical threshold settings. A target was considered positive if a peak fell within the expected size window (target size ±0.5 bp), exceeded the analytical threshold, and met the established peak-shape criteria. Runs with excessive baseline noise or failed size calling were deemed invalid and re-tested once. Samples that remained invalid after re-testing were reported as indeterminate and excluded from subsequent performance calculations. For discordant results between MPCE and NGS, the MPCE assay was repeated once, with NGS serving as the reference method for all performance evaluations.

### 2.4 Limit of detection (LOD) determination

Serial dilutions of positive control DNA were prepared to generate a concentration gradient ranging from 10^6^ to 10 copies/μL. Each concentration level was tested in triplicate. The detection rate for each target gene was determined based on repeated experimental results. The limit of detection (LOD) was defined as the lowest concentration at which all targets were consistently detected in all replicates under the optimized assay conditions.

### 2.5 Cross-reactivity validation

To assess analytical specificity, a panel of 18 non-target microorganisms frequently encountered in routine clinical specimens was tested. The panel included *Streptococcus pneumoniae*, *Candida glabrata*, *Haemophilus influenzae*, *Pseudomonas aeruginosa*, *Acinetobacter baumannii*, *Saccharomyces cerevisiae*, *Legionella pneumophila*, *Aspergillus niger*, *Aspergillus terreus*, *Aspergillus flavus*, *Moraxella catarrhalis*, *Nocardia asteroides*, *Escherichia coli*, *Rhizopus oryzae*, *Aspergillus fumigatus*, *Stenotrophomonas maltophilia*, *Candida albicans,* and *Staphylococcus aureus*. Each organism was processed and analyzed using the same MPCE workflow and interpretation criteria as the target assay.

### 2.6 Repeatability analysis

To evaluate the repeatability of the assay, two hvKp strains (*MDR-hvKp-01* and *MDR-hvKp-02*) and two negative control strains (*Escherichia coli*-01 and *Escherichia coli*-02) were selected. Each strain was tested in 10 replicates under identical experimental conditions. The positive and negative detection rates were subsequently calculated to evaluate assay consistency.

### 2.7 Diagnostic performance evaluation

A total of 60 clinical samples (30 hvKp-positive and 30 hvKp-negative) were analyzed in parallel using both the MPCE assay and NGS. Invalid samples were re-tested once; those that remained invalid were excluded from subsequent performance calculations. For samples with discordant results, the MPCE assay was repeated once as described in Section 2.3, with NGS serving as the reference method. Concordance between the two methods for target gene detection was subsequently evaluated.

### 2.8 Data analysis

All statistical analyses were performed using R version 4.2.2 (R Development Core Team) with a *p*-value < 0.05 considered statistically significant. Agreement between the MPCE assay and NGS was assessed using Cohen’s kappa coefficient. Sensitivity, specificity, positive predictive value (PPV), and negative predictive value (NPV) were calculated using standard formulas, with NGS serving as the reference standard. For genes with 100% specificity, the positive likelihood ratio (LR+) was undefined as a point estimate. In such cases, a conservative lower bound for LR + was estimated using the lower limit of the 95% confidence interval for specificity, based on the standard LR + formula (Akobeng, 2007). For target genes with no positive samples identified by the reference standard, measures of positive diagnostic performance—including sensitivity, LR+, and PPV—were considered not estimable (N/A) due to the absence of a reference-positive population (Fischer et al., 2003).

## 3 Results

### 3.1 Establishment and optimization of MPCE system

A rapid detection system for hvKp was developed using MPCE. This platform allows for the simultaneous identification of five virulence genes, two capsular serotype markers, and seven antibiotic resistance genes, comprising 14 molecular targets. Two internal control genes, rcsA (as the Kp reference gene) and IC (as the system quality control), were also included.

**Figure 1:**
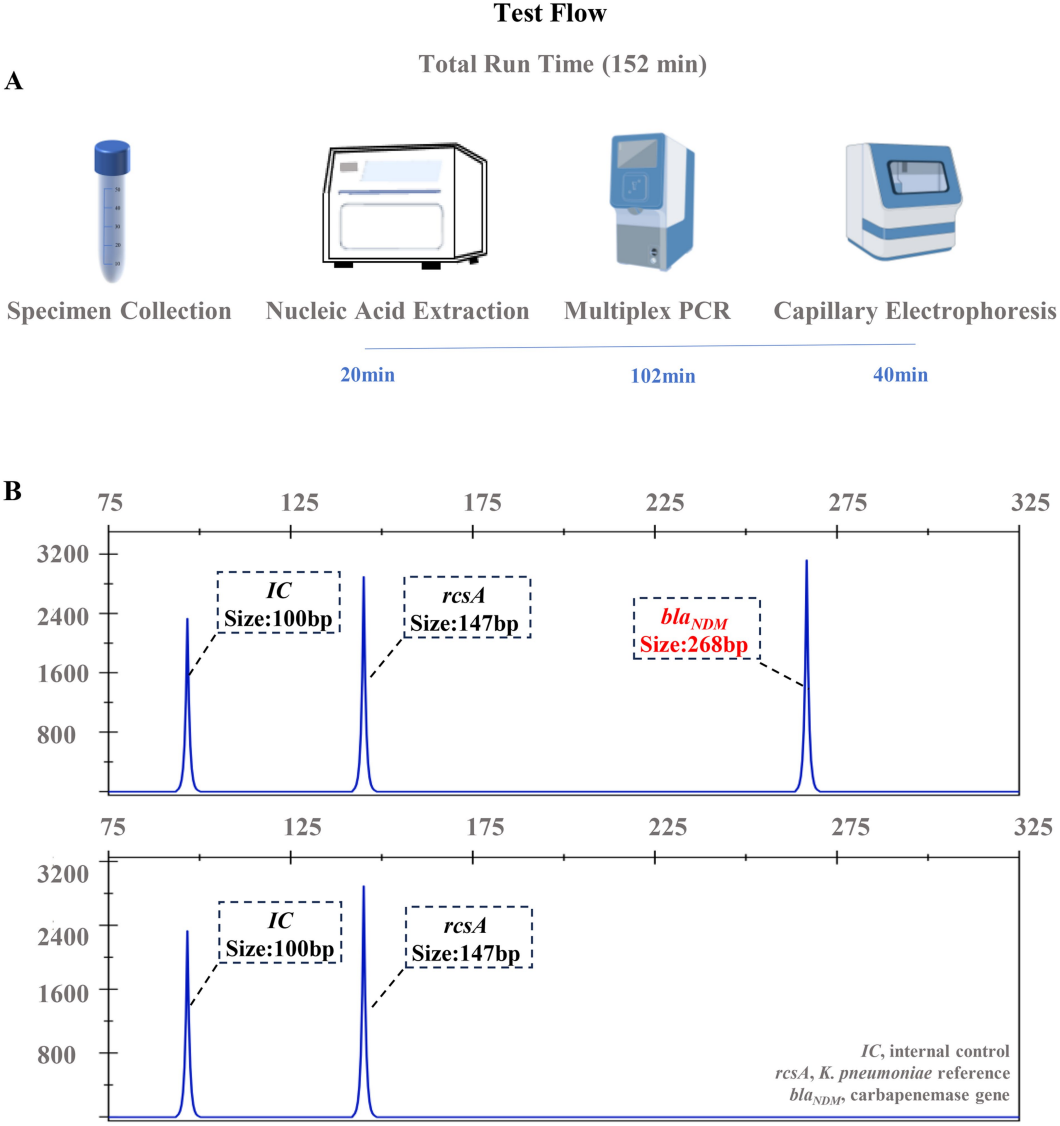
Schematic diagram of the detection process and representative results. **(A)** Workflow of the MPCE assay for hvKp. **(B)** Representative electropherogram showing detection of a blaNDM-positive carbapenem-resistant isolate.

Systematic optimization yielded clearly resolved fluorescent amplicons within the size range of 50 to 400 bp, with single-base resolution, enabling precise differentiation of mixed samples. Figure 1 provides a schematic overview of the detection process (Figure 1A) and demonstrates representative electrophoretograms (Figure 1B), illustrating the specificity of the assay in identifying target genes.

### 3.2 Primer specificity and cross-reactivity assessment

**Figure 2:**
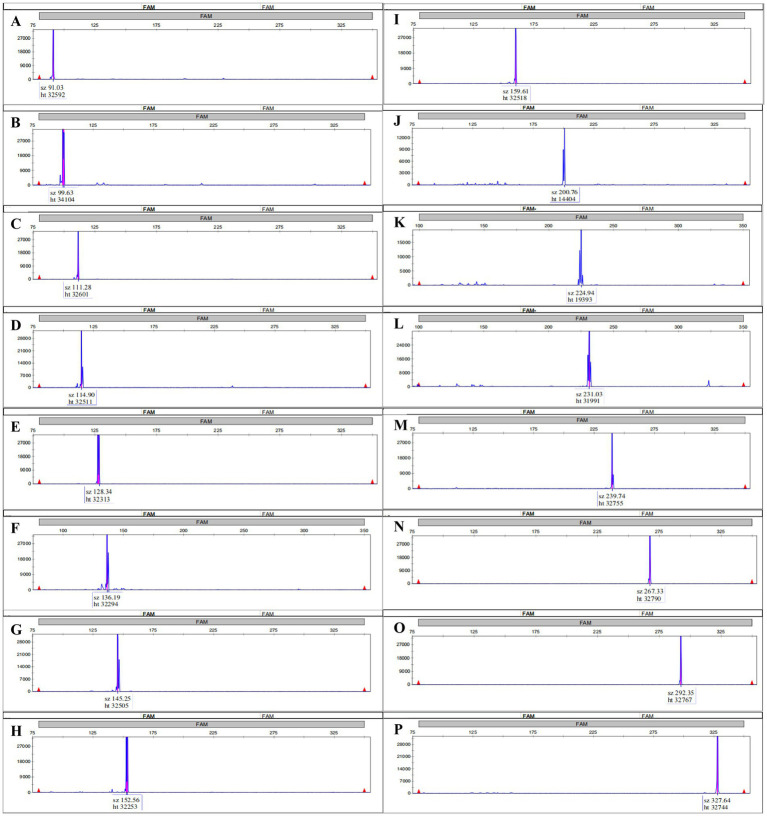
Electropherograms of single-plex MPCE amplification for the 16 target genes. Each panel displays a distinct peak corresponding to the specific amplicon of a target gene. Fragment size (bp) and fluorescence intensity (RFU) are indicated on the *x*- and *y*-axes, respectively, with target genes and their expected amplicon sizes labeled above each peak. **(A)** K2; **(B)** IC; **(C)** *iroB*; **(D)** *peg344*; **(E)** *blaCTX-M*; **(F)** *blaSHV*; **(G)** *rcsA*; **(H)** *blaOXA-23*; **(I)** *mcr-1*; **(J)** *iucA*; **(K)** *rmpA*; **(L)** *rmpA2*; **(M)** K1; **(N)** *blaNDM*; **(O)** *blaOXA-48*; **(P)** *blaKPC*.

**Figure 3:**
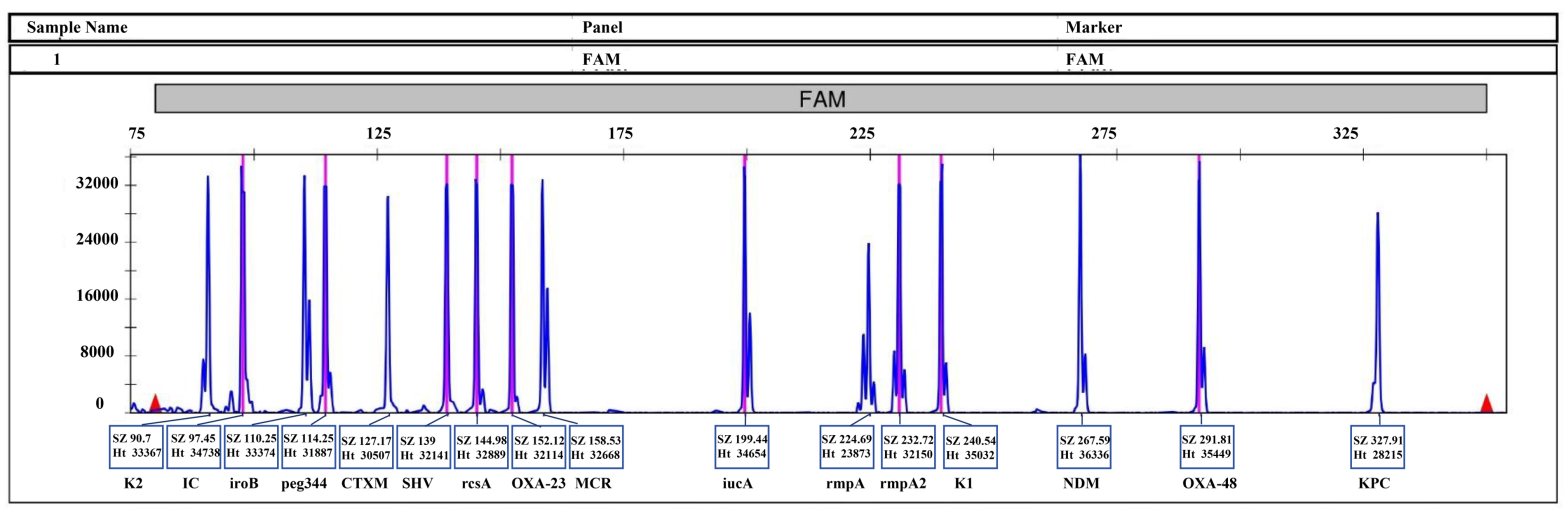
Multiplex MPCE assay for simultaneous detection of 16 target genes. A representative electropherogram illustrates the concurrent amplification of all target genes within a single reaction. Each peak corresponds to a specific amplicon, with gene identity and fragment size (bp) annotated.

The specificity of all primers was validated using DNA templates containing each of the 16 target genes. No cross-amplification was detected, and each primer pair exclusively amplified its corresponding target. Initially, each target was amplified and detected individually via MPCE (Figure 2). Subsequently, DNA from all 16 target genes was mixed and amplified in a single multiplex PCR reaction. Capillary electrophoresis analysis of the products demonstrated that all amplified fragments matched the expected sizes based on the reference sequences (Supplementary Table S1), Figure 3 illustrates the concurrent amplification of all target genes within a single reaction, with each peak corresponding to a specific amplicon; gene identities and fragment sizes (bp) are annotated. These results confirm the high specificity and multiplexing capability of the system.

**Figure 4:**
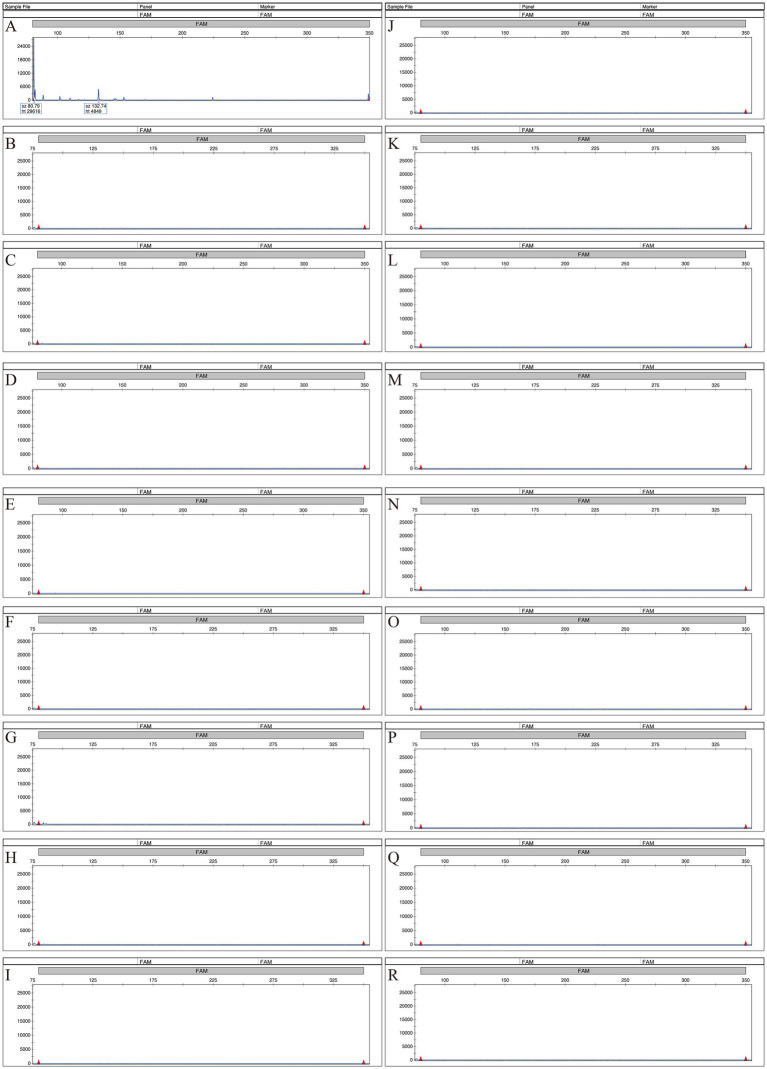
Representative electropherograms from the cross-reactivity evaluation of the MPCE assay using 18 non-target organisms. A panel of 18 non-target microorganisms frequently encountered in clinical specimens was tested. None of the organisms produced peaks exceeding the threshold for any of the 14 assay targets. Representative results are shown for each organism: **(A)** *Streptococcus pneumoniae*, **(B)** *Candida glabrata*, **(C)** *Haemophilus influenzae*, **(D)** *Pseudomonas aeruginosa*, **(E)** *Acinetobacter baumannii*, **(F)** *Saccharomyces cerevisiae*, **(G)** *Legionella pneumophila*, **(H)** *Aspergillus niger*, **(I)** *Aspergillus terreus*, **(J)** *Aspergillus flavus*, **(K)** *Moraxella catarrhalis*, **(L)** *Nocardia asteroides*, **(M)** *Escherichia coli*, **(N)** *Rhizopus oryzae*, **(O)** *Aspergillus fumigatus*, **(P)** *Stenotrophomonas maltophilia*, **(Q)** *Candida albicans* and **(R)** *Staphylococcus aureus*.

To evaluate analytical specificity under clinically relevant background conditions, cross-reactivity testing was performed using 18 non-target microorganisms, including Gram-negative bacteria, Gram-positive bacteria, yeasts, molds, and an actinomycete (Supplementary Table S2). Representative electropherograms are shown in Figure 4. None of the tested organisms produced peaks exceeding the RFU threshold within the predefined size tolerance window for any of the 14 assay targets. Collectively, these findings support the analytical specificity of the MPCE assay within the tested organism panel.

### 3.3 Limit of detection (LOD)

A series of 10-fold serial dilutions (10 to 10^6^ copies/μL) were tested to determine the analytical sensitivity of the assay for all 14 target genes. As summarized in Table 1, the assay reliably detected all targets at template concentrations ≥10^2^ copies/μL using purified control DNA, consistent with the defined LOD criterion. At the lowest concentration (10 copies/μL), the detection rates declined to 57.1% (12/21) for virulence genes and 81% (17/21) for resistance genes. Among the virulence-associated targets, *K2* and *iucA* were not detected at this concentration (0/3), and *K1* and *rmpA2* showed detection in only one (1/3) and two (2/3) replicates, respectively. Among the resistance genes, *blaSHV*, *mcr-1*, *blaKPC* and *blaOXA-48* each demonstrated amplification in two of the three replicates (2/3). The LOD was determined to be 10^2^ copies/μL for all targets, which falls within the typical concentration range observed in clinical samples (10^3^ to 10^6^ copies/μL).

**Table 1:** Limit of detection (LOD) of the MPCE assay for 14 target genes.

Target gene	10^6^ copies/μL	10^5^ copies/μL	10^4^ copies/μL	10^3^ copies/μL	10^2^ copies/μL	10 copies/μL
*iucA*	3/3	3/3	3/3	3/3	3/3	0/3
*iroB*	3/3	3/3	3/3	3/3	3/3	3/3
*peg344*	3/3	3/3	3/3	3/3	3/3	3/3
*rmpA*	3/3	3/3	3/3	3/3	3/3	3/3
*rmpA2*	3/3	3/3	3/3	3/3	3/3	2/3
*CTX-M*	3/3	3/3	3/3	3/3	3/3	3/3
*SHV*	3/3	3/3	3/3	3/3	3/3	2/3
*OXA-23*	3/3	3/3	3/3	3/3	3/3	3/3
*MCR*	3/3	3/3	3/3	3/3	3/3	2/3
*NDM*	3/3	3/3	3/3	3/3	3/3	3/3
*KPC*	3/3	3/3	3/3	3/3	3/3	2/3
*OXA-48*	3/3	3/3	3/3	3/3	3/3	2/3
*K1*	3/3	3/3	3/3	3/3	3/3	1/3
*K2*	3/3	3/3	3/3	3/3	3/3	0/3

Each concentration was tested in triplicate. The LOD was defined as the lowest concentration yielding 100% detection across all replicates, which was 10^2^ copies/μL for all targets.

### 3.4 Assay repeatability

**Figure 5:**
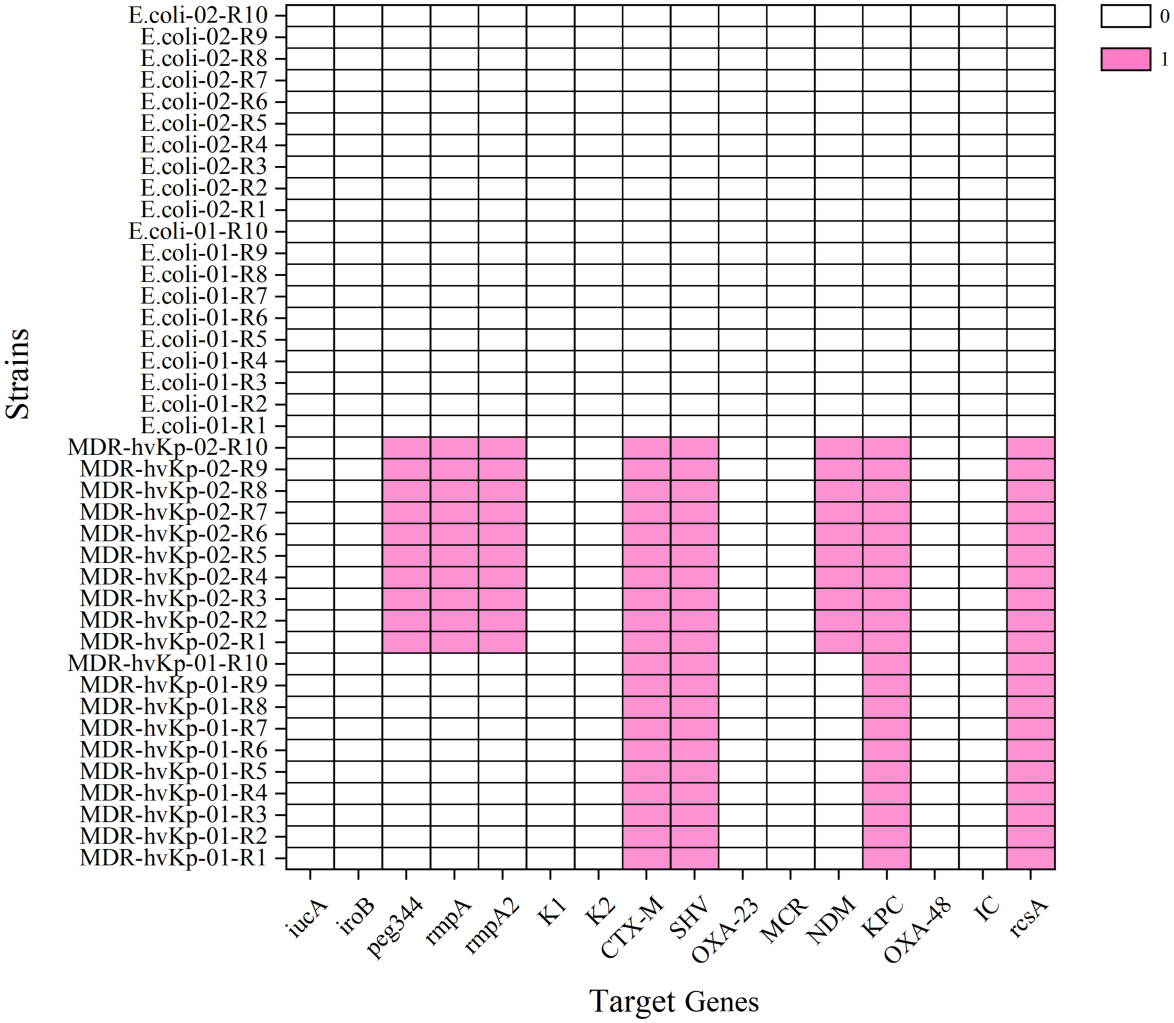
Assessment of assay repeatability. The assay was evaluated across ten replicates using two *MDR-hvKp* strains (*MDR-hvKp-01* and *MDR-hvKp-02*) and two negative control strains (*Escherichia coli*-01 and *Escherichia coli*-02). Both positive and negative detection results demonstrated 100% agreement across all replicates, confirming the excellent repeatability of the assay.

A total of ten independent replicate assays were conducted for each of two MDR-hvKp strains (*MDR-hvKp-01* and *MDR-hvKp-02*) and two negative control strains (*Escherichia coli-01* and *Escherichia coli-02*). All 10 replicate runs produced concordant qualitative results across all reportable targets, indicating high repeatability of the assay under the tested conditions (Figure 5). These findings confirm the high reproducibility of the assay in accurately distinguishing *K. pneumoniae* strains harbouring diverse resistance and virulence gene profiles.

### 3.5 Application to clinical samples

The diagnostic accuracy of the MPCE assay compared to the NGS reference standard is summarized in Table 2. Overall, the assay demonstrated excellent performance across all target genes, with kappa values indicating substantial to almost perfect agreement (*κ* = 0.659–1.000, *p*<0.001). Sensitivity was 100% for the majority of genes (11/14), with the exception of *iucA* (85.71%; 95% CI: 63.66%–96.95%) and *blaNDM* (92.59%; 95% CI: 75.71%–99.09%). Specificity was also high, reaching 100% for 10 genes and ranging from 86.67 to 98.31% for the remaining four. For genes with perfect specificity, LR + could not be calculated as point estimates due to the absence of false positives. To provide clinically meaningful values, conservative lower bounds were derived from the lower limits of the specificity 95% CIs, ranging from 9.49 to 16.50, indicating strong rule-in capability. Negative likelihood ratios (LR-) were 0 or near 0 for most genes, confirming excellent rule-out value. The *bla*OXA-48 gene had no positive cases by NGS, thus sensitivity and LR + were not estimable (N/A), though specificity was 100% (95% CI: 92.60–100%).

**Table 2:** Diagnostic performance of the MPCE assay compared to NGS for 14 target genes in clinical samples (*n* = 60).

Target gene	Sensitivity (%) (95% CI)	Specificity (%) (95% CI)	LR+(95% CI)	LR-(95% CI)	PPV (%) (95%CI)	NPV (%) (95% CI)	Kappa
*iucA*	85.71 (63.66–96.95)	100 (90.97–100)	> 9.49^a^	0.14 (0.05–0.41)	100 (81.47–100)	92.86 (80.52–98.50)	0.886
*iroB*	100 (73.54–100)	100 (92.60–100)	> 13.51^a^	0	100 (73.54–100)	100 (92.60–100)	1.000
*peg344*	100 (63.06–100)	96.15 (86.79–99.53)	26.00 (6.68–101.20)	0	80.00 (44.39–97.48)	100 (92.89–100)	0.870
*rmpA*	100 (47.82–100)	100 (93.51–100)	> 15.41^a^	0	100 (47.82–100)	100 (93.51–100)	1.000
*rmpA2*	100 (63.06–100)	92.31 (81.46–97.86)	13.00 (5.07–33.33)	0	66.67 (34.89–90.08)	100 (92.60–100)	0.762
*CTX-M*	100 (88.43–100)	86.67 (69.28–96.24)	7.50 (3.01–18.68)	0	88.24 (72.55–96.70)	100 (86.77–100)	0.867
*SHV*	100 (78.20–100)	88.89 (75.95–96.30)	9.00 (3.94 – 20.57)	0	75.00 (50.90–91.34)	100 (91.19–100)	0.800
*OXA-23*	100 (2.50–100)	98.31 (90.91–99.96)	59.00 (8.45–411.92)	0	50.00 (1.26–98.74)	100 (93.84–100)	0.659
*MCR*	100 (39.76–100)	98.21 (90.45–99.96)	56.00 (8.03–390.62)	0	80.00 (28.36–99.49)	100 (93.51–100)	0.880
*NDM*	92.59 (75.71–99.09)	96.97 (84.24–99.92)	30.56 (4.42–211.14)	0.08 (0.02–0.29)	96.15 (80.36–99.90)	94.12 (80.32–99.28)	0.899
*KPC*	100 (80.49–100)	100 (91.78–100)	> 12.16^a^	0	100 (80.49–100)	100 (91.78–100)	1.000
*OXA-48*	N/A	100 (94.04–100)	N/A^b^	N/A^b^	N/A^b^	100 (94.04–100)	N/A^b^
*K1*	100 (2.50–100)	100 (93.94–100)	> 16.50^a^	0	100 (2.50–100)	100 (93.94–100)	1.000
*K2*	100 (2.50–100)	100 (93.94–100)	> 16.50^a^	0	100 (2.50–100)	100 (93.94–100)	1.000

NGS was used as the reference standard for all calculations. Each target gene was assessed in 60 clinical samples (30 hvKp-positive and 30 hvKp-negative).

CI, confidence interval; LR+, positive likelihood ratio; LR-, negative likelihood ratio; PPV, positive predictive value; NPV, negative predictive value; N/A, not applicable.

^a^For genes with 100% specificity, LR+ could not be calculated as a point estimate due to the absence of false positives; the values shown (> X) represent conservative lower bounds derived from the lower limits of the specificity 95% confidence intervals.

^b^For blaOXA-48, no positive cases were identified by the reference standard (NGS); thus, sensitivity, LR+, LR–, PPV, and kappa were not estimable.

## 4 Discussion

This study established a rapid MPCE assay for the simultaneous detection of core virulence genes, capsular serotypes, and antimicrobial resistance genes in hvKp. The MPCE system demonstrated high sensitivity, specificity, and repeatability, offering a reliable technical platform for the rapid clinical screening of hvKp and its associated resistance profiles. Given the emerging threat of multidrug-resistant hvKp, this system represents a valuable tool for both the rapid diagnosis of infections and the molecular epidemiological characterization of drug-resistant hvKp strains.

The analytical sensitivity, assessed through serial dilution experiments, confirmed that the MPCE assay could stably detect all target genes at template concentrations ≥ 10^2^ copies/μL, achieving a 100% detection rate. A slight reduction in detection rates was observed for certain low-abundance targets at approximately 10 copies/μL. This LOD adequately covers the pathogen load typically observed in clinical samples, which generally ranges from 10^3^ to 10^6^ copies/μL. The observed performance of the MPCE assay is comparable to that of other rapid molecular diagnostics, demonstrating sensitivity comparable to that of the LAMP assay recently reported by Cai et al. (2025). However, although certain isothermal amplification or recombinase-mediated assays, such as RAA and LAMP, may achieve a lower LOD for individual targets (Yan et al., 2023; Wang et al., 2024). The MPCE assay enables multi-gene synchronous detection while maintaining a sensitivity of 10^2^ copies/μL. This integrated approach thereby avoids operational complexity and substantial sample consumption associated with running multiple discrete reactions. Nevertheless, because clinical matrices such as sputum and blood can contain PCR inhibitors (Cai et al., 2018), the practical sensitivity in inhibitor-rich specimens may differ and warrants further evaluation.

Specificity assessment revealed no cross-reactivity within the tested panel of 18 non-target organisms, and the MPCE genotyping results were highly concordant with NGS in this study. The single-base resolution of capillary electrophoresis enabled clear discrimination between highly homologous targets such as *rmpA* and *rmpA2*, addressing a limitation of conventional gel-based multiplex PCR systems. Nevertheless, broader validation against diverse *Enterobacterales* collections, including closely related Klebsiella species, will be important to further substantiate analytical specificity. The MPCE system also demonstrated excellent repeatability across replicate runs, supporting the robustness of multiplex peak calling. Although multiplex PCR coupled with capillary electrophoresis is an established approach (Wang et al., 2024; Jiang et al., 2017), the present assay integrates 14 clinically relevant targets into a single reaction with high-resolution sizing, enabling concurrent virulence-serotype-resistance profiling. While recent work by Kulkarni et al. reported a multiplex assay targeting carbapenemase genes in hvKp (Kulkarni et al., 2025), our panel extends this concept by simultaneously incorporating ESBL genes (*bla_CTX-M_*, *bla_SHV_*) and the plasmid-mediated colistin resistance gene (*mcr-1*), providing a more comprehensive resistance profile. Because MPCE relies on capillary electrophoresis instrumentation, it may be most readily implemented in laboratories with existing capillary electrophoresis capacity.

The MPCE assay demonstrated excellent diagnostic accuracy for detecting clinically significant antimicrobial resistance genes, with near-perfect sensitivity and specificity for most targets and substantial to almost perfect agreement with NGS (*κ* = 0.659–1.000). These findings align with previous reports highlighting the utility of multiplex PCR-based methods for rapid resistance gene surveillance (Qin et al., 2024; Wada et al., 2025). For genes with 100% specificity, LR + could not be calculated as point estimates due to the absence of false positives. However, conservative lower bounds—derived from the lower limits of the specificity 95% CIs—all exceeded 7.8, with most above 10, a conventional threshold for strong diagnostic evidence (Kulkarni et al., 2025). This indicates that a positive result from the MPCE assay provides robust confirmation of gene presence, minimizing false-positive risks. Conversely, negative likelihood ratios (LR–) were 0 or near 0 for most genes, confirming excellent rule-out capability valuable for antimicrobial stewardship (Kulkarni et al., 2025). The *bla*OXA-48 gene had no positive cases by NGS, precluding estimation of sensitivity and LR+; however, 100% specificity (95% CI: 92.60–100%) ensures reliable negative reports, consistent with previous observations that low-prevalence genes may require larger cohorts for full performance assessment (Wada et al., 2025). Study limitations include small sample sizes for low-prevalence genes, resulting in wide confidence intervals, and evaluation using cultured isolates rather than direct clinical specimens. Despite these constraints, the MPCE assay proves to be a highly accurate tool for rapid resistance gene detection, supporting its integration into clinical microbiology workflows.

This study has several limitations. The validation was performed in a single-center setting using a medium-sized cohort comprising both clinical isolates and contrived samples. Future prospective, multi-center validation studies encompassing diverse geographical regions and various sample types such as blood, bronchoalveolar lavage fluid, liver aspirates, and cerebrospinal fluid are warranted. Moreover, the predictive accuracy of different resistance genes for phenotypic resistance exhibited variability in *Klebsiella pneumoniae* (Wang et al., 2026). This study focused on genotypic detection, and the concordance between genotype and phenotype was not evaluated; therefore, phenotypic testing remains essential for confirming resistance. Furthermore, in the dynamic landscape of carbapenem-resistant hypervirulent *K. pneumoniae* (CR-hvKp) evolution, characterized by the continual emergence of novel plasmid variants, resistance determinants, and truncation sites (Pu et al., 2023), it is essential to establish an iterative process for regularly updating primer and marker panels to ensure the assay’s continued wide coverage. Despite these limitations, the MPCE assay serves as a rapid screening and monitoring tool that provides results within hours, which can be complemented by NGS for detailed strain lineage analysis and epidemiological investigations when needed.

## 5 Conclusion

The MPCE-based assay established in this study offers an efficient, accurate, and reliable platform for the rapid screening of hvKp and its associated antimicrobial resistance genes. This system represents a valuable resource for enhancing antimicrobial resistance surveillance and epidemiological investigations of hvKp. With future multi-center validation and standardization of technical workflows, the MPCE assay has the potential to become a powerful asset in clinical microbiology laboratories, ultimately contributing to antimicrobial stewardship and infection control efforts.

## Data Availability

The original contributions presented in the study are included in the article/Supplementary material, further inquiries can be directed to the corresponding author/s.
