# Supplementary material for: A rapid detection system for core virulence and resistance genes in hypervirulent Klebsiella pneumoniae using multiplex fluorescence PCR-capillary electrophoresis
Source: Front Microbiol. 2026 Apr 20;17:1798786. doi: 10.3389/fmicb.2026.1798786 (PMC13136247; doi:10.3389/fmicb.2026.1798786)
Supplement: Supplementary file 1 [file Table_1.DOCX]

**Supplementary Table S1. Primers used in the MPCE assay.**

| **No.** | **Target Gene** | **Primer** | **Primer Sequences（5'-3'）** | **Fluorescent Label** | **Amplicon Size/bp** |
| --- | --- | --- | --- | --- | --- |
| **1** | ***iucA*** | **iucA-F** | **ATGGCTATTCCCGCTGCAC** | **5'-FAM** | **200** |
|  |  | **iucA-R** | **GAGTTGGTCAGGCGCACG** |  |  |
| **2** | ***iroB*** | **iroB-F** | **CCCTCCGCTCGGAGTCAT** | **5'-FAM** | **110** |
|  |  | **iroB-R** | **GAACGCGTTACGCCTTTGAT** |  |  |
| **3** | ***peg344*** | **peg344-F** | **CCATTATTAATCATTATCGCATGGG** | **5'-FAM** | **114** |
|  |  | **peg344-R** | **TCATTAGMGAGAAAGGAATGGCAAA** |  |  |
| **4** | ***rmpA*** | **rmpA-F** | **CCTCTGCTTCATATTACATTTGAAGGAGTA** | **5'-FAM** | **224** |
|  |  | **rmpA-R** | **CATAGATGTCATAATCACACCCTTTAGG** |  |  |
| **5** | ***rmpA2*** | **rmpA2-F** | **CACACTACCTCTGGTTTATATTACGTATGAAGGCTCG** | **5'-FAM** | **232** |
|  |  | **rmpA2-R** | **CATAGATGTCATAATCACACCCTTTAGG** |  |  |
| **6** | ***CTX-M*** | **CTXM-F** | **CRATGTGCAGYACCAGTAAAGT** | **5'-FAM** | **128** |
|  |  | **CTXM-R** | **TTCSGCAATCGGATTRTAGTTAAC** |  |  |
| **7** | ***SHV*** | **SHV-F** | **GCAAAAAGGCAGTCAATCCTG** | **5'-FAM** | **137** |
|  |  | **SHV-R** | **TCGCCGGTCAGCGAAAAACAYCTTGC** |  |  |
| **8** | ***OXA-23*** | **OXA23-F** | **AAGGTCATTTACCGCTTGGGA** | **5'-FAM** | **152** |
|  |  | **OXA23-R** | **TCCAATTTCAGCATTACCGAAACCAATACG** |  |  |
| **9** | ***MCR*** | **MCR-F** | **ATCGCTGTCGTGCTCTTTGG** | **5'-FAM** | **159** |
|  |  | **MCR-R** | **GAGCATGGTCGTATCATAGACCG** |  |  |
| **10** | ***NDM*** | **NDM-F** | **AATGTCTGGCAGCACACTTCCTAT** | **5'-FAM** | **268** |
|  |  | **NDM-R** | **TCGACAACGCATTGGCATAA** |  |  |
| **11** | ***KPC*** | **KPC-F** | **AACCATTCGCTAAACTCGAACAG** | **5'-FAM** | **327** |
|  |  | **KPC-R** | **ACTCCTTCAGCAACAAATTGGC** |  |  |
| **12** | ***OXA-48*** | **OXA48-F** | **CGCTCCGATACGTGTAACTTATTG** | **5'-FAM** | **292** |
|  |  | **OXA48-R** | **TGAACACCAAGTCTTTAAGTGGGAT** |  |  |
| **13** | ***K1*** | **K1-F** | **TTTAGGTATAGCCGATTCTGATGT** | **5'-FAM** | **240** |
|  |  | **K1-R** | **CTCGCGAGTAAATAAGCAATAGATG** |  |  |
| **14** | ***K2*** | **K2-F** | **ACTCCATTTTAGCTTTTGCCATAA** | **5'-FAM** | **90** |
|  |  | **K2-R** | **GTTCCTACGCTCCAACTTCTTTG** |  |  |
| **15** | ***rcsA*** | **rcsA-F** | **TTGCCTTTATGCGACGATACC** | **5'-FAM** | **147** |
|  |  | **rcsA-R** | **CAGTGTAGGGCAGTTAACTTTACCG** |  |  |
| **16** | ***IC*** | **IC-F** | **TAGGTTGTTTAAAGGAATCTGGG** | **5'-FAM** | **100** |
|  |  | **IC-R** | **CGACCTAATGGTACTATGGGAGTC** |  |  |

Note: *Abbreviations: MPCE, multiplex fluorescence PCR-capillary electrophoresis; FAM, carboxyfluorescein; HPLC, high-performance liquid chromatography.*

Mixed bases: R = A/G; Y = C/T; S = G/C; M = A/C. These degenerate primers were designed to cover sequence variations among different variants of the target genes
