# Supplementary material for: A rapid detection system for core virulence and resistance genes in hypervirulent Klebsiella pneumoniae using multiplex fluorescence PCR-capillary electrophoresis
Source: Front Microbiol. 2026 Apr 20;17:1798786. doi: 10.3389/fmicb.2026.1798786 (PMC13136247; doi:10.3389/fmicb.2026.1798786)
Supplement: Supplementary file 2 [file Table_2.DOCX]

| **Supplementary Table S2. Cross-reactivity testing of the MPCE assay against non-target microorganisms** | | | |
| --- | --- | --- | --- |
| **No.** | **Organism** | **Category** | **MPCE Result** |
| **1** | *Streptococcus pneumoniae* | Gram-positive bacterium | Negative |
| **2** | *Candida glabrata* | Yeast | Negative |
| **3** | *Haemophilus influenzae* | Gram-negative bacterium | Negative |
| **4** | *Pseudomonas aeruginosa* | Gram-negative bacterium | Negative |
| **5** | *Acinetobacter baumannii* | Gram-negative bacterium | Negative |
| **6** | *Saccharomyces cerevisiae* | Yeast | Negative |
| **7** | *Legionella pneumophila* | Gram-negative bacterium | Negative |
| **8** | *Aspergillus niger* | Mold | Negative |
| **9** | *Aspergillus terreus* | Mold | Negative |
| **10** | *Aspergillus flavus* | Mold | Negative |
| **11** | *Moraxella catarrhalis* | Gram-negative bacterium | Negative |
| **12** | *Nocardia asteroides* | Actinomycete | Negative |
| **13** | *Escherichia coli* | Gram-negative bacterium | Negative |
| **14** | *Rhizopus oryzae* | Mold | Negative |
| **15** | *Aspergillus fumigatus* | Mold | Negative |
| **16** | *Stenotrophomonas maltophilia* | Gram-negative bacterium | Negative |
| **17** | *Candida albicans* | Yeast | Negative |
| **18** | *Staphylococcus aureus* | Gram-positive bacterium | Negative |

Note: A negative result indicates that no peaks exceeding the analytical threshold were observed within the expected size windows for any of the 14 target genes
